# Focal Adhesion-Chromatin Linkage Controls Tumor Cell Resistance to Radio- and Chemotherapy

**DOI:** 10.1155/2012/319287

**Published:** 2012-06-18

**Authors:** Katja Storch, Nils Cordes

**Affiliations:** ^1^OncoRay-National Center for Radiation Research in Oncology, Medical Faculty Carl Gustav Carus, Dresden University of Technology, Fetscherstraße 74, 01307 Dresden, Germany; ^2^Department of Radiation Oncology, Medical Faculty Carl Gustav Carus, Dresden University of Technology, Fetscherstraße 74, 01307 Dresden, Germany

## Abstract

Cancer resistance to therapy presents an ongoing and unsolved obstacle, which has clear impact on patient's survival. In order to address this problem, novel *in vitro* models have been established and are currently developed that enable data generation in a more physiological context. For example, extracellular-matrix- (ECM-) based scaffolds lead to the identification of integrins and integrin-associated signaling molecules as key promoters of cancer cell resistance to radio- and chemotherapy as well as modern molecular agents. In this paper, we discuss the dynamic nature of the interplay between ECM, integrins, cytoskeleton, nuclear matrix, and chromatin organization and how this affects the response of tumor cells to various kinds of cytotoxic anticancer agents.

## 1. Introduction

Resistance to radiotherapy, chemotherapy, and novel molecular drugs still represents one of the major obstacles in cancer therapy [1–3]. Limited effectiveness of therapy inevitably results in progressive disease or recurrence, thereby reducing the chance of cure for the patients. Phenotypically, two types can be distinguished: pretherapeutically existing and acquired resistances [4, 5]. Acquired resistance to irradiation is not known, but anticancer drugs, both conventional and molecular, frequently induce defense mechanisms [6–8]. To optimize the efficacy of cytotoxic agents, it is necessary to ameliorate drug delivery to the tumor and to better understand the underlying molecular mechanisms causing the resistance or evolving the defense process [5, 9].

In order to address the latter, we and others focused on a particular cellular substructure called focal adhesion (FA) [10–17]. FAs are membrane areas, which cells employ to interact with the surrounding extracellular matrix (ECM) via integrin adhesion receptors [10, 17–22]. Due to their multiprotein composition including growth factor receptors, signaling, and adapter proteins, FAs are huge hubs for signaling downstream to control critical cell functions such as cell survival, proliferation, differentiation, and invasion [11, 13, 14, 18, 20, 22–30]. The highly complex interplay between all of these signaling molecules secures homeostasis of single cells as well as of tissues in the context of responses to external signals from the microenvironment.

In tumor cells, according to the hallmarks of cancer [31], the proper physiological communication with the extracellular space is massively disturbed as a consequence of gene mutations and epigenetic modifications. Despite tumor growth-driving gene mutations, malignant cells often retain a high degree of susceptibility to certain extracellular factors [13, 31–34]. Prime examples are microenvironmental signals induced by cell adhesion to the ECM, adhesion to neighbouring cells, and growth factor receptor-ligand interactions, which all contribute to tumor progression and resistance to cytotoxic injury resulting from chemotherapeutic drugs and irradiation [12, 32, 35].

Keeping these facts in mind, a lot of effort was put in the improvement of *in vitro* models that best reflect *in vivo* growth conditions [11, 33, 36–40]. Since the advent of ECM-based 3D cell culture assays, a large body of evidence has suggested that conventional monolayer models do not reflect the complexity of tissues, phenotypes of cells, and modifications in transcriptome, proteome, phosphoproteome, protein-protein interactions, and signal transduction as the 3D models [11, 40–53].

From the therapeutic point of view, flat monolayer cell cultures contain an ECM-integrin-cytoskeleton connection very different from 3D grown cells [22, 40, 45, 46, 50, 51, 54, 55]. Moreover, cell growth in 3D ECM shows additional features like 3D multicellular spheroid growth [38, 56, 57]. Common to all 3D conditions is that the responsiveness to extracellular signals, drug, and radiation sensitivity as well as the physical forces between ECM and cytoskeleton for controlling chromatin organization and gene expression is very different from cells cultured in 2D. In this paper, we discuss cell-adhesion-mediated radio- and chemoresistance in the context of signaling and interplay between ECM, integrins, cytoskeleton, nuclear matrix, and chromatin organization.

## 2. Microenvironmental Signals Including Integrin Signaling Regulate Cellular Radio- and Chemosensitivity

Next to genetic alterations, the microenvironment plays an important role for carcinogenesis, tumor progression, and development of therapy-resistant phenotypes [31]. A closer look at the initiators and promoters of this multistep process suggests that a combination of both extra- and intracellular events commonly occurs to activate proto-oncogenes and deactivate tumor suppressor genes [31]. With regard to carcinogenesis, the particular reasons for cancer development can only be assumed in the minority of cases. Exploring a “mature” tumor provides a picture of the accumulated alterations in the various molecular determinants, which maintain unlimited growth and cause both existing and *de novo* therapy resistance mechanisms. In addition to the aforementioned genetic modifications, various soluble and structural microenvironmental factors like cytokines, chemokines, growth factors, and ECM essentially contribute to anticancer therapy defense mechanisms [13, 58–63].

Importantly, the ECM has structural, signaling, and storage functions. Thus, cells communicate with the surrounding ECM by mechanotransduction, by integrin-mediated adhesion, and by growth factor release and subsequent binding to their cognate receptors [21, 64–68]. For the role of mechanotransduction, only one issue has been evidently shown: changes in ECM stiffness induce perturbations of normal cell physiology preparing the ground for malignant transformation [42, 50, 69–71]. Open questions are, for example, how changes in ECM expression pattern of tumors impact on tumor cell behavior or how therapy-related alterations in tumor structure influence integrin-ECM interactions and intracellular signaling. For cell-adhesion-mediated radioresistance (CAM-RR) and cell-adhesion-mediated drug resistance (CAM-DR), integrins play critical roles [59, 72, 73].

Integrins are transmembrane receptors consisting of an *α* and a *β* chain. The 18 *α* and 8 *β* subunits form 24 known *αβ*-heterodimers dependent on cell type and function [20]. Integrin signals are transferred via the cell membrane in both directions. The binding activity of integrins is regulated from the inside and is called inside-out signaling; the interaction of integrins with ECM proteins for signal transduction into the cell is called outside-in signaling [10, 17–22]. These interactions essentially contribute to the regulation of various cellular functions like proliferation, survival, adhesion, differentiation, migration of cells, and tissue integrity [29, 41, 74–77].

For many years, it remained unknown how integrin signaling mediates tumor cell resistance. Well known were increased survival and reduced apoptosis in irradiated or drug-treated tumor cells of varying origin like head and neck, lung, pancreas, glioma, colon, breast, cervix, prostate, myeloma, and leukemia [58, 78–83]. But which signaling cascades do transmit these biochemical prosurvival signals? Physiologically, a large set of signal transduction and adapter molecules assembles at the cytoplasmic integrin domain upon integrin binding to ECM [17, 20]. Formation of mature FAs is critical for robust cell adhesion to ECM as well as accessibility to the intracellular signaling network for optimized regulation of key cellular processes [10, 16, 17]. For this signaling, integrins and growth factor receptors need to cooperatively and mutually interact [84]. Both adapter and nonreceptor bound signaling proteins are recruited to integrin or growth factor receptor tails upon activation. Through proteins such as focal adhesion kinase (FAK), small GTPases of the Rho family, PI3K/Akt, JNK, and ERK as well as the ternary protein complex consisting of integrin-linked kinase (ILK), PINCH1 and alpha-parvin (IPP), talin, alpha-actinin, and vinculin, biochemical signals are transferred as a result of integrin/RTK commitment [14, 15, 17, 85]. Despite prosurvival signaling, it remains to be solved what exact impact morphology has on cell survival. ECM-integrin-actin cytoskeletal and cell-cell-intermediate filament connections determine cell morphology, which consequently define function and integrity of single cells and tissues [33, 34, 37, 42, 62, 86]. A variety of molecules involved in these interactions have been shown to be altered in cancer. For example, integrins are overexpressed in many human cancers originating from the head and neck region [87], lung [88], prostate [89], ovary [90], and breast [91], while E-cadherin, as one of the key cell-cell contact proteins, is frequently reduced in its expression or absent [87, 92, 93].

These expression changes are highly likely to impact on tumor cell behavior. We know that this physical linkage between ECM and cytoskeleton via integrins is crucially involved in translating mechanical into chemical signals and in controlling cell morphology [37, 64, 68, 71, 94]. *In vivo*, the ECM determines the shape and stiffness of tissues [34, 62, 95]. Under conventional cell culture conditions, *ex vivo* cultured cells grow attached to artificial surfaces like cell culture plastic. Missing physiological cell-matrix contacts, as optimally met in a 3D environment, has dramatic impact on cell shape and cellular behavior *in vitro* [12, 13, 37, 41, 69, 86, 96]. A large number of studies demonstrated that 2D cultured cells lose important features of their original phenotype due to severe genetic, epigenetic, and signal transduction changes [42, 48, 71, 97]. As this is similarly true for normal and cancer cells, one might realize that tumor cells have a preserved susceptibility for external signals originating from the ECM or soluble extracellular factors. Obviously, these facts are contrary to observations demonstrating an independency from external input signals by autonomous activation of intracellular pathways or activating mutations in proto-oncogenes leading to anchorage-independent growth [13, 31, 32]. Doubtless, mutation-driven, constitutively activated oncogenes overrun antiproliferative signals from the outside, but the myriad of additional stimuli affecting the cells is very well perceived and processed.

Taking these features into account, one can easily imagine that ECM and integrins contribute to the regulation of the cellular reaction to genotoxic injury. Onoda et al. found that nonlethal irradiation of melanoma cells induces alphaIIb/beta 3 integrin upregulation and increased adhesion to fibronectin [98]. Further studies corroborated these findings in a variety of normal and transformed human cell lines [59, 72, 78, 99–101]. However, clinically important is the fact that integrin-mediated cell adhesion to the surrounding ECM confers resistance to ionizing radiation, cytotoxic drugs, and molecular agents [59, 72, 102–104]. In addition to CAM-DR [59] and CAM-RR [72], a new paradigm was entitled “Environment-Mediated Drug Resistance” by Meads and colleagues (EMDR) [60]. Intriguingly, these three phenomena have been confirmed in irradiated or drug-treated cells from various tumor entities like glioma, leukemia, and melanoma as well as carcinomas of the pancreas, lung, and head and neck [18, 25, 28, 59, 72, 105–107].

Besides increased cell survival, ECM attachment prolonged radiogenic G2/M cell cycle arrest [103, 108] and reduced the number of residual DNA double-strand breaks (DSBs) and lethal chromosomal aberrations [40]. Also apoptotic cell death of small cell lung cancer cells was diminished under adhesion to laminin, fibronectin, or collagen type IV upon treatment with cytotoxic drugs [83].

Based on these findings, efforts are undertaken to uncover the underlying mechanisms and identify the cellular mediators and determinants involved in CAM-RR and CAM-DR. To elucidate therapeutic possibilities, small interfering RNA (siRNA) knockdown and antibody-mediated integrin inhibition are evaluated in different tumor cell lines with promising effects. In breast carcinoma, head and neck carcinoma, glioma, and leukemia cells, beta1 integrin targeting resulted in enhanced radiosensitivity and apoptosis [26, 27, 47, 80, 102, 109]. The pseudokinase ILK was clearly identified as antisurvival molecule in an attempt to classify the pro- and antisurvival function(s) of molecules acting downstream of integrins in cancer cells exposed to radiotherapy (reviewed in [110, 111]). Amongst others, this prosurvival group of molecules consists of FAK, JNK1, Akt1, PINCH1, and Caveolin-1 [55, 104, 112–117]. For example, overexpression of FAK protects 3D grown head and neck squamous cell carcinoma (HNSCC) cells from radiation-induced cell death [118], while siRNA-mediated silencing or pharmacological inhibition of FAK increases the radiosensitivity of different tumor cell lines from pancreatic cancer [112], breast cancer, colorectal cancer [119], and HNSCC [45, 55]. Furthermore, human melanoma cells become more sensitive to the chemotherapeutic agent 5-fuorouracil when FAK expression is downregulated [120]. In pancreatic cells, a reduction of FAK expression using microRNA for RNA interference leads to decreased FAK phosphorylation and repressed chemoresistance to gemcitabine [121]. Another interesting key player in this field is the LIM domain-containing particularly interesting new cysteine-histidine-rich 1 protein (PINCH1). Recent work from our group showed that knockdown of PINCH1 diminished the chemo- and radioresistance of diverse human carcinoma cells *in vitro* and *in vivo *[43, 122]. Mechanistically, PINCH1 was identified as novel Akt1 regulator by serving as platform for a regulatory interaction between protein phosphatase 1*α* (PP1*α*) and Akt1 [43]. According to the radiosensitization upon PINCH1 depletion, increased numbers of radiogenic DSBs were found in PINCH1 knockdown cell cultures indicating a role of PINCH1 in DNA repair processes [122]. On the basis of these findings, identification and targeting of molecules such as FAK or PINCH1 that critically regulates the cytotoxic drug and radiation response of tumor cells is a promising concept to overcome radio- and chemoresistance of tumor cells to improve cancer patient survival.

Additionally and of high importance for the current concepts of multimodal therapies, integrin-mediated cell-ECM interactions confer reduced efficacy of novel molecular agents/small molecules. In HNSCC, Eke and colleagues showed that adhesion to fibronectin attenuates the antiproliferative effect of a potent pharmacological epidermal growth factor receptor (EGFR) tyrosine kinase inhibitor [104]. Recent findings provide evidence for an essential role of ILK for EGFR targeting in HNSCC [113] and that EGFR overexpression mediates hypersusceptibility to the anti-EGFR antibody cetuximab in 3D grown HNSCC cell lines in a FAK-dependent manner [55].

In summary, integrin-mediated adhesion to ECM protects cancer cells from varying types of cell death intended by radiotherapy, chemotherapy, and molecular drugs. Monotherapeutic targeting of integrins and intracellular signaling molecules to overcome adhesion-mediated resistance is already part of first clinical trials [13]. Cilengitide, a peptide potently blocking *ανβ*3 and *ανβ*5 integrin, is currently under evaluation in clinical phase II trials in glioblastoma and other malignancies [123, 124]. Identification of novel potential cancer targets and evaluation of targeted approaches against these targets require extensive examination and, most importantly, consideration and usage of preclinical tumor models, which best reflect clinical circumstances.

## 3. The Impact of Focal Adhesion-Chromatin Linkage on Tumor Resistance against Irradiation and Cytotoxic Drugs

Regardless of normal or malignant cells, extracellular factors control critical cellular functions like survival, proliferation, and differentiation in a tissue-specific context [23, 37, 71, 97]. Studies using *ex vivo* cell cultures show the loss of morphological and functional properties in an artificial environment such as cell culture plastic as compared to ECM scaffolds [38, 69, 71]. Interesting studies in diverse tumor cell lines and normal cells showed that 3D growth in a matrix modifies gene and protein expression, cell survival, proliferation, differentiation, and metabolism in comparison to conventional 2D monolayer cell cultures [40, 42, 43, 46, 48, 116]. In line with these findings, osteosarcoma cells are protected against doxorubicin treatment [125] and head and neck and non-small-cell lung cancer cells display a reduced radiation sensitivity when grown in a 3D matrix in contrast to 2D [11, 40]. Beside these effects on cell survival upon cytotoxic injury, 3D growth conditions result in differential gene expression [126]. Global reorganization of chromatin through varying ECM compositions has been shown to be accompanied by changes in gene expression [94, 95, 127, 128]. Early work from Barcellos-Hoff and colleagues indicated that although polarized monolayers are formed, mammary epithelial cells fail to express milk proteins in 2D [23]. In 3D laminin-rich ECM, however, they formed alveolar-like structures with a central lumen and secreted milk proteins like casein [48, 49, 71, 95]. In this context, genes with ECM-responsive elements (EREs) were identified and helped to explain how the ECM participates in the regulation of gene expression [94, 128–130]. Furthermore, the pattern of gene expression is controlled by chromatin organization, which in turn is regulated by posttranslational modifications, that is, acetylation, phosphorylation, and methylation of nucleosomal histones [131, 132]. 

By integrating the above into an ECM-integrin-actin-nuclear membrane-nuclear matrix scenario, the ECM serves as one of the most powerful determinants of chromatin organization and gene expression [94, 130]. Concurrently, histones are upregulated as shown in 3D grown neuroblastoma cells [133] and tumor spheroids of melanoma cells [134], and histone acetylation is decreased to cooperatively control gene expression [46, 86, 135]. These highly dynamic actions are facilitated by histone acetyltransferases (HAT) and histone deacetylases (HDAC) [136, 137]. Gene expression in less condensed, euchromatic DNA regions is associated with histone hyperacetylation, while transcriptional repression occurs in more dense, heterochromatic DNA regions, by deacetylation [132, 138]. Recent own data demonstrate that growth in 3D ECM scaffolds decreases the levels of histone H3 acetylation in line with enhanced expression of the heterochromatin protein HP1*α* indicating a higher amount of heterochromatin [40]. Additionally, Le Beyec et al. highlighted the impact of cell-shape-induced changes in histone acetylation [46]. Cells cultured on polyHEMA showed a round cell morphology that led to histone deacetylation as consequence of changes in cell morphology but not adhesion [46]. These observations indicate that modifications in cell morphology impact on gene expression and thereby fundamentally determine tissue homeostasis and cellular responsiveness to external stress signals in a microenvironment-specific context [69].

**Figure 1:**
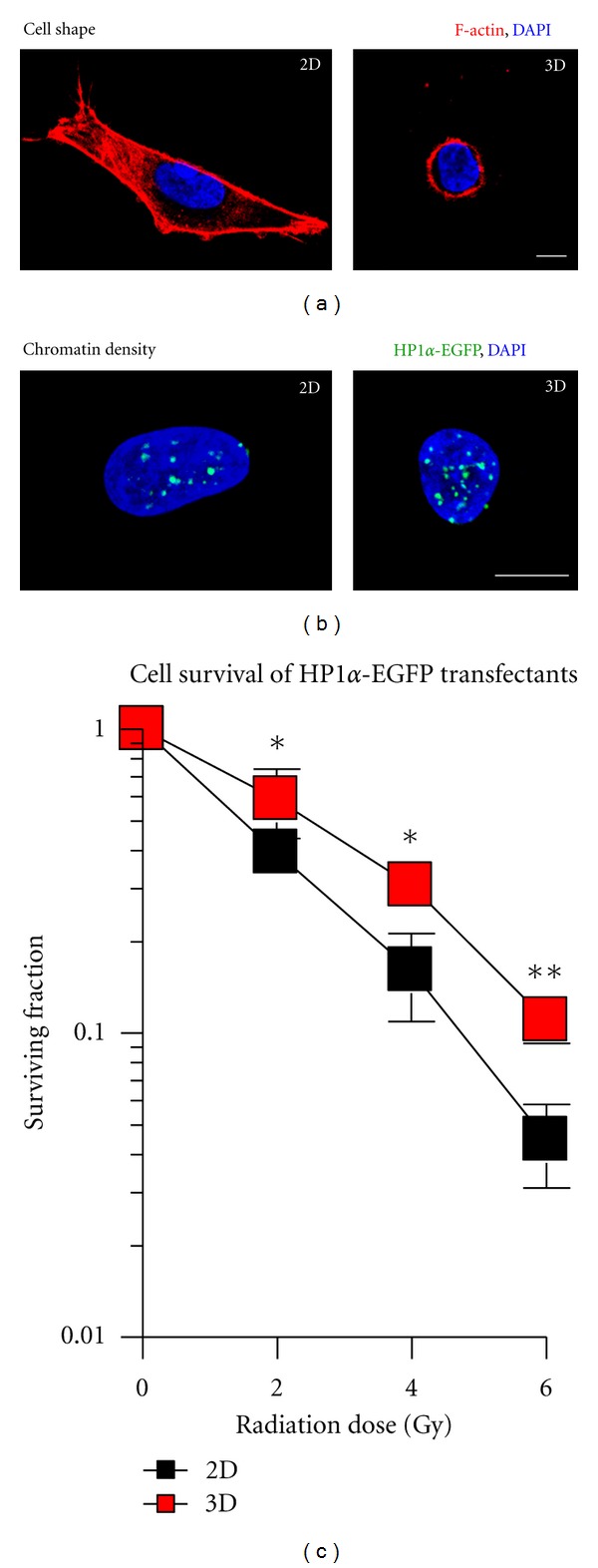
Cell morphology, HP1*α*-EGFP distribution and clonogenic radiation survival of cells grown under three-dimensional (3D) growth conditions. (a) Comparison of cell morphology under 2D and 3D growth conditions (green; DAPI, blue, F-actin, red). (b) Fluorescence images of 2D and 3D grown A549 cells expressing HP1*α*-EGFP fusion protein. Images were acquired using laser scanning microscopy. (c) 2D and 3D clonogenic radiation survival of HP1*α*-EGFP expressing A549 cells irradiated with single doses of X-rays (0–6 Gy). Means ± SD and Student's *t*-test comparing 3D *versus* 2D conditions. *P < 0.05; **P < 0.01; *n* = 3; bar, 10 *μ*m.

Hence, it is most likely that cells cultivated in 3D show also differences in pathways of DNA repair after treatment with DNA-damaging agents in comparison to 2D. Little is known about the distribution of radiogenic DSBs within areas with different chromatin condensation status. With regard to the increased radiation survival caused by reduced numbers of residual DSBs and a lower number of chromosomal aberrations, 3D cell growth induces larger amounts of heterochromatin in comparison to 2D (Figure 1) [40]. Furthermore, these data show DSB localization in eu- and heterochromatic DNA regions to be similar in 3D and tumor xenografts. Conversely to this 1 : 1 distribution, 2D cells show a 2 : 1 eu- to heterochromatin DSB distribution [40]. These results underline the findings that 3D cell culture models better mimic the *in vivo* situation than conventional 2D monolayers.

**Figure 2:**
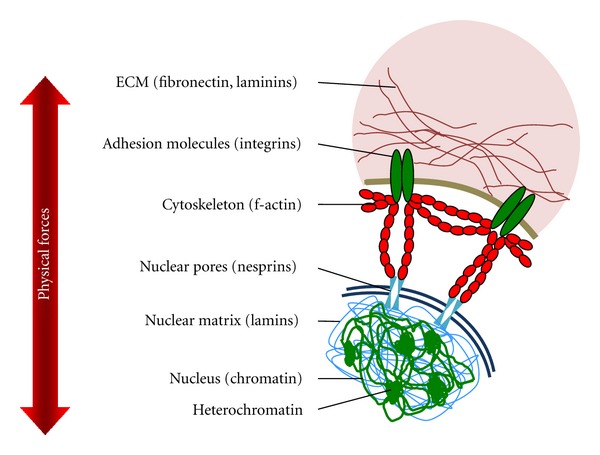
Schematic of the interplay between extracellular matrix (ECM), cytoskeleton, and nuclear matrix and the physical forces that affect cell morphology and chromatin organization.

How are ECM and nuclear matrix linked? Cytoskeletal filaments physically bridge between integrins or other cell adhesion molecules and the nuclear matrix including chromatin structures (Figure 2) [17, 18, 64, 127, 139]. Thus, both cell-matrix-activated signal transduction and mechanical forces sensed at the surface promote structural rearrangements in the cytoplasm and in the nucleus [68, 127, 140]. The linkage between cytoskeletal filaments and the nuclear matrix was identified as a complex termed linker of nucleoskeleton and cytoskeleton (LINC) and contains nesprins, sun, and lamin proteins [68, 141–143]. Nesprins 1 and 2 are nuclear membrane proteins that bind actin filaments and interact with sun proteins at the inner nuclear membrane. To control nuclear organization and gene function according to external stimuli, lamin proteins, which are connected with the inner nuclear membrane, form a nuclear scaffold that can bind chromatin directly or indirectly via other nuclear proteins [142, 144].

Through this complex interplay between ECM, integrins, cytoskeleton and nuclear matrix, many changes such as genome reorganization and differential gene expression, alterations in cell morphology, and integrin-mediated signal transduction occur in response to microenvironmental factors [13, 23, 33, 129]. Importantly, this focal adhesion-chromatin linkage contributes to existing and acquired therapy resistance in cancer. An increased understanding of the underlying molecular mechanisms and the implementation of better translational cancer models will assist our efforts to optimize and personalize cancer therapy.
